# Is membrane homeostasis the missing link between inflammation and neurodegenerative diseases?

**DOI:** 10.1007/s00018-015-2038-4

**Published:** 2015-09-24

**Authors:** Natalia Sánchez de Groot, Marc Torrent Burgas

**Affiliations:** 1 Laboratory of Molecular Biology (Medical Research Council), Francis Crick Avenue, Cambridge, CB2 0QH UK; 2 Department of Biochemistry and Molecular Biology, Universitat Autònoma de Barcelona, 08193 Bellaterra, Spain; 3 Vall d’Hebron Research Institute (VHIR), Passeig Vall d’Hebron 119-129, 08035 Barcelona, Spain

**Keywords:** Protein aggregation, Neurodegenerative disease, Neuroinflammation, Membrane, Microglia

## Abstract

Systemic inflammation and infections are associated with neurodegenerative diseases. Unfortunately, the molecular bases of this link are still largely undiscovered. We, therefore, review how inflammatory processes can imbalance membrane homeostasis and theorize how this may have an effect on the aggregation behavior of the proteins implicated in such diseases. Specifically, we describe the processes that generate such imbalances at the molecular level, and try to understand how they affect protein folding and localization. Overall, current knowledge suggests that microglia pro-inflammatory mediators can generate membrane damage, which may have an impact in terms of triggering or accelerating disease manifestation.

## Introduction

Neurodegenerative diseases are familial and sporadic conditions characterized by the progressive dysfunction of the nervous system [1, 2]. In some cases, the origins of these diseases can be related to destabilizing mutations in proteins, which become less stable and more prone to aggregating or interacting with undesired partners [3]. In these examples, neurodegenerative conditions have a genetic, inheritable component and individuals develop them at an earlier age. One of the most studied cases is Alzheimer’s disease (AD), where mutations in the amyloid precursor protein (APP) can lead to the aggregation of the processed amyloid beta peptide (Aβ), generating characteristic plaques in the brain [4]. Furthermore, in amyotrophic lateral sclerosis (ALS) and Parkinson’s disease (PD), mutations in superoxide dismutase and α-synuclein, respectively, may cause the aggregation of these proteins and affect the function of motor neurons, causing tremors and muscle paralysis [5, 6].

However, many patients suffering from late onset neurodegenerative diseases lack a predefined genetic background. Indeed, it is widely recognized that such disorders are multifactorial and associated with both an individual genome and environmental conditions [7].

Although the molecules and signaling pathways involved in the inflammatory response have been widely characterized, it is still unclear how they influence the development of neurodegenerative diseases and are linked to environmental challenges. In this paper, we review how inflammatory mechanisms can have an impact on membrane homeostasis and how this may perturb the key proteins associated with neurodegenerative disorders.

## Inflammation in neurodegenerative diseases

Though direct infection or hyper-sensitivity to foreign proteins may cause an intrinsic inflammation, the brain is also vulnerable to damage in response to systemic inflammation as infiltration of immune cells and mediators can lead to profound structural and functional changes [8].

The blood–brain barrier (BBB) isolates the central nervous system (CNS) from the circulating blood, creating a privileged environment. While this is required to maintain brain homeostasis, it does not mean that the brain is depleted of immune cells [9]. Also, the CNS is not completely isolated from blood circulation [10] as cytokines may: (1) by-pass the BBB at the circumventricular organs and mobilize resident macrophages; (2) activate the brain endothelial cells and signal the microglia within the parenchyma; (3) activate the sensory afferents of the vagus nerve communicating with neurons and, finally, (4) be actively transported by the endothelium across the BBB. Microglia cells, as resident macrophages of the CNS, play a central role in the innate immune response [11, 12]. In the absence of damage, resting microglia have a ramified morphology and constantly survey the environment in order to identify potentially harmful signals that require a response [13]. When this happens, the microglia take on an amoeboid morphology, changing the expression of surface receptors and secreting pro- and anti-inflammatory mediators (e.g., chemokines and cytokines), recruitment factors, and chemicals such as reactive oxygen species (ROS). In this state, the microglia cells are described as activated.

Bacteria can activate microglia and promote memory impairment in young mice through a mechanism that involves interleukin-1, and the high-mobility group protein B1 [14]. For example, a single sublethal injection of lipopolysaccharide (LPS) can impair behavior and memory in mice, reduce the proliferation of neural stems cells and induce microglia invasion and activation to the hippocampus [15]. Severe systemic inflammation, such as sepsis, also leads to an increased production of the nuclear factor κB [16] that changes the microglia phenotype [17]. Most interesting, there is convincing evidence that increased amounts of inflammatory biomarkers are observed before the clinical onset of dementia. For example, Kuo and co-workers [18] found that an increase in C-reactive protein in serum was increased 5 years before the clinical onset of dementia. In another case, Buchhave and co-workers showed that TNF receptors TNFR1 and TNFR2 are also associated with the development of dementia 4–6 years before to disease manifestation [19].

In this section, we aim to review how inflammation is connected to the most studied neurodegenerative diseases. We also highlight recent studies that support the notion that triggering the brain immune system, either during early embryonic stages or by chronic activation at a later age, can promote and/or accelerate the development of neurodegenerative disorders.

### Inflammation in Alzheimer’s disease

Alzheimer’s disease is a multifactorial, neurodegenerative illness that is manifest as a cognitive impairment and behavioral disorder [20]. Histopathological analyses of the brains of patients with the condition reveal nerve and synapse loss, but also two characteristic lesions: plaques containing aggregated Aβ peptide and tau neurofibrillar tangles [21].

A significant number of aged individuals with AD suffer from more than one systemic disease [22] suggesting that systemic inflammation might be a risk factor for AD. In fact, systemic inflammation and acute infections have been associated with cognitive decline in patients with AD [23, 24] and genome-wide analyses suggest that several genes that increase the risks for sporadic Alzheimer’s disease encode factors that regulate inflammatory reactions [25].

In a recent study, Krstic and colleagues developed a model to demonstrate that viral infections are related to late onset AD. The team treated isogenic mice with either the viral mimic polyriboinosinic-polyribocytidilic acid (PolyI:C) or a placebo, and measured neuropathological changes during aging [26]. Only the mice treated with the RNA analog developed amyloid plaques and experienced significant impairments in their working memory at older ages. These results suggest not only that inflammation and infection can increase the probability of developing AD, but also that environmental conditions can trigger neurodegenerative disorders irrespective of the genetic background.

The same authors suggest that during healthy aging misfolded and aberrant proteins are directed to budding structures called intracellular varicosities, which are later extruded, engulfed and degraded by microglia cells [27]. According to the authors’ model, under chronic inflammatory stress, microglia cells become permanently activated and cannot properly remove these deposits. Thereafter, axonal varicosities swell and accumulate APP, jeopardizing axonal transport. Moreover, activated microglia secrete pro-inflammatory mediators and reactive oxygen species that contribute to neuronal damage. Ultimately, the aberrant processing of APP (misfolding stress) and excessive neuronal damage (oxidative stress) promote the formation of amyloid plaques that are released upon neuron death.

Chronic inflammatory stimuli are also involved in tau phosphorylation and tangle formation. Kitazawa and colleagues found an increase in the former at specific sites in 3xTg-AD transgenic mice after they were injected with LPS, which also exacerbates pre-tangle pathology in a cyclin kinase 5-dependent mechanism [28]. Although in their experiments the progressive activation of microglia was found to correlate with the onset of fibrillar aggregates, the causal relationship between immune activation, tau phosphorylation and fibrillation is still unclear. In another study, Bhaskar and his colleagues observed that intraperitoneally administered LPS induced Iba1^+^ activated microglia promoting tau hyperphosphorylation in non-transgenic mice [29]. They also noted that mice lacking the fractalkine receptor CX3CR1 displayed enhanced tau phosphorylation and aggregation, as well as behavioral impairments. When LPS activates microglia, it induces the release of fractalkine that binds to the microglia G-coupled fractalkine receptor, downregulating the activation and dampening the toxic effects of the activated microglia [29]. In fact, knocking out CX3CR1 in various mouse models of AD worsens the phenotype [30] and aggravates cognitive deficits [31]. As a result, it is possible that chronic neuroinflammation leads to the microglia being in a permanently activated state that is insensitive to fractalkine signaling, thus endangering neurons.

### Inflammation in Parkinson’s disease

Parkinson’s disease is the second most common neurodegenerative disorder after AD, and causes a slow and progressive degeneration of dopaminergic neurons in the substantia nigra and a later degeneration in the central cortex [32]. Patients affected by this disease have characteristic intracellular protein inclusions called Lewy bodies that contain α-synuclein, among other proteins. The origin of these inclusions and the causes of neuronal loss are not fully understood. Patients with PD have an increased level of pro-inflammatory mediators in their cerebrospinal fluid (including TNF-α, IL-1β and IL-6) and a presence of microglial cells in the substantia nigra [33]. These changes are associated with the progression of the disease, but it is not known whether they are involved in the pathological process or are a consequence of neuronal degeneration.

Experiments in animal models have revealed that the injection of LPS leads to an accumulation of α-synuclein and the progressive degeneration of the dopamine nigrostriatal system, causing motor impairments [34]. Those responsible for this research determined that neuroinflammation induced by LPS generates damage caused by the S-nitrosylation/nitration of mitochondrial proteins. These events are followed by the progressive degeneration of dopaminergic neurons in the nigrostriatal system [34]. Recent studies suggest that cytokine IL-1 plays a central role in mediating the functional changes induced by LPS, but the precise mechanism by which microglial activation occurs and leads to motor neuron impairment remains to be determined [35].

As observed in AD, fractalkine receptor CX3CR1-knockout mouse models of PD also reveal the worsening of the phenotype [30], suggesting that the modulation of inflammation by fractalkine signaling can protect against microglial neurotoxicity. Interestingly, Nash and his co-workers found that soluble fractalkine is capable of reducing the dopaminergic neuron loss caused by human α-synuclein over-expression [36].

### Inflammation in amyotrophic lateral sclerosis

Amyotrophic lateral sclerosis is one of the most common late onset neurogenerative diseases, with a prevalence of ∼5 per 100,000 individuals. It is characterized by the selective deterioration of motor neurons, which leads to the progressive atrophy of skeletal muscles [37].

Autopsies of ALS patients have revealed that active demyelination and neurodegeneration are present in those with marked brain inflammation [38]. Recently, Frakes and colleagues found that NF-κB signaling pathway is activated in ALS, predominantly in microglia [39]. Moreover, the deletion of microglia NF-κB signaling rescues motor neurons in mice with ALS by inhibiting pro-inflammatory activation [39]. NF-κB is a protein complex that plays a central role in regulating the immune response to infection, and is also activated in the microglia cells of patients suffering from AD and PD [40, 41].

In 2009, Saijo and his co-workers discovered an orphan receptor called Nurr1 that functions as a key component of a negative feedback loop in both microglia and astrocytes. Nurr1 acts by recruiting the CoREST corepressor to NF-κB target genes, regulating NF-κB turnover and restoring its expression to a basal state. In this context, the absence of Nurr1 makes cells susceptible to the toxicity caused by mutations in superoxide dismutase 1 (SOD1), which is an enzyme linked to ALS [42]. Interestingly, Nurr1 is also related to other neurodegenerative pathologies, and promotes exaggerated and prolonged inflammatory responses that accelerate the loss of dopaminergic neurons in response to α-synuclein overexpression [43]. Furthermore, the number of Nurr1-expressing cells significantly declines in an age-dependent manner that is concomitant with increased Aβ accumulation [44].

### Inflammation in Huntington’s disease

Other neurodegenerative diseases have also been associated with inflammation. An example is Huntington’s disease (HD), where the aggregation of huntingtin causes motor impairments and neuropsychiatric disorders [45].

Similar to other neurodegenerative pathologies, cells expressing mutant huntingtin with an expanded polyglutamine (polyQ) repeat have elevated NF-κB activity [46]. Moreover, in cortico-striatal slices and primary neuronal culture models, the microglia are localized in the vicinity of neurons expressing mutant huntingtin genes and have increased levels of the pro-inflammatory cytokine IL-6 [47].

Taken together, all of this evidence highlights the fact that neuroinflammation is a general hallmark of neurodegenerative diseases, and that a permanent activation of the brain immune system could increase the probability of developing such conditions. Even more relevant, the literature suggests that neurodegenerative diseases may share inflammatory pathways, including the activation of the NF-κB pathway and the deregulation of fractalkine signaling.

In the next section, we attempt to elucidate how inflammation is connected to both protein aggregation and cell damage.

## Inflammation and membrane damage

Membranes are transcendental structures in organisms, because they compartmentalize the cell and organize cellular processes. The cell membrane is a lipid bilayer that envelops the entire cell, physically separating the internal milieu from the extracellular environment. The cell membrane acts a barrier, but also enables the transport of molecules and ions and allows cell-to-cell communication.

Lipid bilayers must be impermeable to molecules and ions from the surrounding environment, but, at the same time, they need to be fluid enough to allow dynamic processes like protein traffic within the membrane [48]. This delicate balance can be easily disturbed by changes in lipid structure and composition. This occurs, for example, due to oxidative damage or when proteins and chemicals affect the membrane recycling cycle. Overall, these processes modify the bilayer fluidity and alter the membrane organization, i.e., they globally disturb membrane homeostasis [49]. This homeostasis is critical for ensuring the curvature of the membrane, correct receptor signaling, endocytosis, exocytosis and organelle biogenesis.

When human microglia are activated during inflammation, they produce reactive species of oxygen and nitrogen (ROS) to kill invading pathogens. However, ROS can also affect surrounding host cells, inducing neurotoxicity [50, 51]. In these situations, as the most external part of the cell, membranes can sustain a great deal of damage from these ROS. Peroxidation processes target lipids within the plasma membrane, in particular, polyunsaturated phospholipids that modify membrane fluidity. Such changes can affect the interaction of proteins and lipids across the membrane. For example, macrophages treated with hydrogen peroxide increase both the gel phase and raft domains, reducing overall membrane fluidity [52]. Similar effects have also been described in other cell types, such as T-lymphocytes and endothelial cells [53, 54]. Activated microglia also secrete pro-inflammatory mediators like cytokines, interleukins and antimicrobial peptides. These molecules can interact with the cell membrane, promoting a structural rearrangement. Antimicrobial peptides, for instance, can redistribute cholesterol, reduce membrane fluidity and create pores, damaging the membrane [55–57].

Lipid homeostasis is critical for protein activity, cell membrane recycling and accurate signaling. In fact, lipid composition alterations in the aged brain can have a dramatic effect on maintaining synaptic functions, including membrane fusion processes, neurotransmitter receptor dynamics and survival/death signaling pathways [58].

An uncontrolled inflammatory response can, therefore, damage neurons by directly disturbing the cell membrane homeostasis. Yet can this have an effect on the formation of protein aggregates and the deposits observed in neurodegenerative diseases? There are two important reasons suggesting that this may be the case:

(1) All the known proteins related to brain neurodegenerative diseases need to bind to membranes to exert their functions:

i. In AD, APP is an integral membrane protein, and the tau association with the plasma membrane is crucial for its correct phosphorylation [59].

ii. In PD, α-synuclein plays a role in lipid transport and synaptic membrane biogenesis [60].

iii. In ALS, the binding of superoxide dismutase 1 (SOD1) to the membrane is required for adequate copper distribution [61].

(2) Several observations suggest that membranes provide a singular environment for amyloid-like aggregates:

i. The diffusion of proteins in membranes occurs in two dimensions, which enhances the probability of protein–protein interactions [62].

ii. Membranes support the better growth of amyloid fibrils [63]. Also the presence of lipid rafts may provide a singular environment to regulate fibril formation and aggregation [64].

iii. Oligomers generally interact with membranes and cause membrane damage (e.g., pore formation) [65].

Accordingly, inflammation may cause an imbalance in membrane homeostasis and, in doing so, affect the aggregation process of the proteins that are related to well-known neurodegenerative diseases. In the following sections, we review how this can be linked to specific neurodegenerative pathologies.

### Membrane perturbation in Alzheimer’s disease

APP is in equilibrium when it is between its native form and the cleavage between different secretases [66]. It can be cleaved by α-secretase, leading to a free APP-sα peptide and a membrane-bound CTFα in the non-amyloidogenic cascade. This process takes place in the non-lipid raft portion of the membrane, and the species produced are non-pathogenic. However, lipid raft-associated APP can be recruited in the endosomes and cleaved by β-secretase (also known as BACE), generating a soluble peptide known as APP-sβ and a membrane-bound peptide called CTFβ. The latter is then processed by the lipid raft-associated γ-secretase to generate toxic Aβ species. These endosomes can be later degraded in lysosomes or re-exported to the membrane through the membrane recycling process that secretes Aβ fragments to the extracellular medium.

**Fig. 1:**
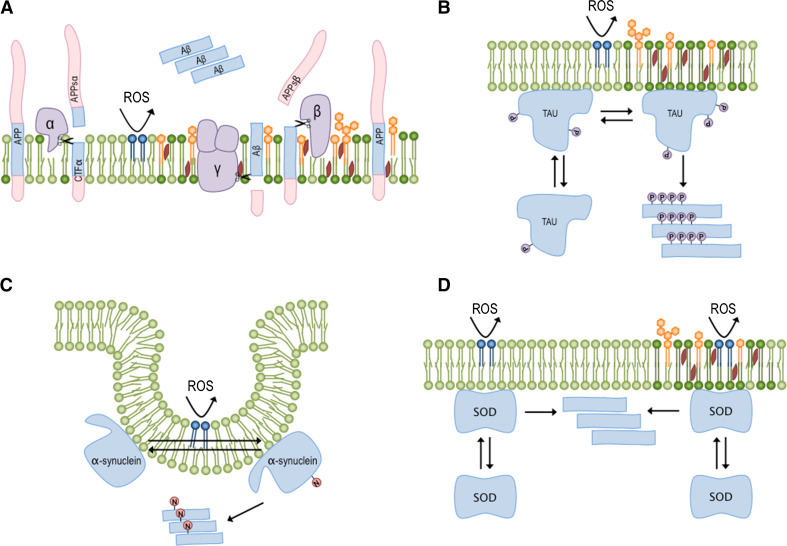
Inflammation can disturb the membrane and promote protein aggregation. Activated microglia cells produce peptides, proteins and reactive species of oxygen and nitrogen that can perturb membrane homeostasis. The binding of peptides and the oxidation of phospholipids can increase the gel phase and raft domains, triggering protein aggregation and disease. **a** APP is in equilibrium between its native and cleaved forms, the latter of which is generated by the action of different secretases (*purple*). In the non-lipid raft portion, which is abundant in unsaturated phospholipids (*light green*), cleavage by α-secretase produces soluble APP and membrane-bound CTFα. However, in terms of lipid rafts, where the membrane domains are rich in cholesterol (*red*), glycosphingolipids (*orange*) and saturated lipids (*dark green*), APP cleavage by β-secretase can generate APP-sβ and CTFβ. The latter peptide can subsequently be processed by γ-secretase to produce the aggregation prone Aβ peptide (*blue*). **b** Changes in membrane composition can modify the phosphorylation pattern (*purple circles*) of the tau protein and promote its aggregation propensity. Aβ aggregates and tau neurofibrillar tangles are characteristic of AD. **c** Alterations in the distribution of α-synuclein between different membrane domains can trigger a self-assembling process, which can be accelerated by lipid peroxidation (*dark blue*). α-synuclein oligomers can damage neuron membranes and promote the development of PD. **d** Superoxide dismutase (SOD) can also bind to different membrane domains in different compositions, and its distribution between them can modulate its aggregation propensity. Specifically, an increase in the content of saturated fatty acids and oxidized lipids can support SOD-oligomerization, thereby promoting the development of ALS

In this scenario, as Aβ formation is raft dependent, the situations that shift the distribution of APP to lipid rafts could increase Aβ production, triggering AD [67]. For example, a decrease in membrane fluidity due to lipid oxidation could lead to the APP accumulating in the gel phase, increasing APP cleavage by γ-secretase and toxic Aβ peptide levels (Fig. 1a).

Much evidence supports the notion that a decrease in membrane fluidity could enhance the production of Aβ and accelerate its aggregation:

(1) Saturated phospholipids and gel phase membranes support the growth of amyloid intermediates better [63, 68].

(2) Cholesterol exposure increases Aβ production by clustering APP and BACE together [69, 70].

(3) AD predominantly affects the cerebral cortex and hippocampus, which is a region of the brain that is significantly enriched in cholesterol when compared to other areas such as the cerebellum [71].

(4) Diets lower in cholesterol and saturated lipids reduce the risk of developing AD [72].

Membrane homeostasis imbalances can also be related to tau aggregation. Maas and colleagues used a microsphere separation process to isolate plasma membrane-associated tau and reported that it was differentially phosphorylated when compared with cytosolic tau [73]. More recently, Hernandez and her co-workers analyzed tau phosphorylation after the incubation of SHSY-5Y cells with Aβ peptide [74]. The authors observed both an increase in Tyr18 phosphorylated tau after 2 min of treatment and a higher association with lipid rafts. After 10 min, they began to detect Ser396 and Ser404 phosphorylated tau, which became more intensively associated with lipid rafts, suggesting a correlation between phosphorylation and raft association. In addition, several authors have reported that tau aggregation is modulated by the bilayer lipid composition [75, 76]; specifically, negatively charged and oxidized phospholipids can increase its aggregation [76, 77]. Consequently, changes in membrane dynamics can affect protein folding and phosphorylation, affecting the location, function and aggregation of tau (Fig. 1b). Indeed, tau intermediate aggregates may promote neuronal damage through membrane disruption [76].

### Membrane perturbation in Parkinson’s disease

α-Synuclein is a protein that is neuropathologically related to PD, and promotes the loss of dopamine-producing neurons in the mid-brain [78]. It is widely accepted that α-synuclein forms toxic oligomeric conformations that disrupt the synaptic function and eventually lead to neuronal death [79].

The binding of α-synuclein to membranes is fundamental for its natural function, but this interaction can also start a nucleation process that generates seeds that can later grow by incorporating cytosolic monomers [80].

Unfortunately, the precise membrane location of α-synuclein is still controversial. Fortin and her co-workers demonstrated that lipid rafts settle the synaptic localization of α-synuclein [81]. Meanwhile, in a recent study, Pranke and colleagues revealed that α-synuclein does not localize widely to the plasma membrane, but only to vesicular clusters closely associated with it [82]. As a result, α-synuclein distribution between the different lipid phases of the plasma membrane may impact its function and its aggregation properties (Fig. 1c).

Lipid peroxidation in the brain substantia nigra has been reported in patients suffering from PD [83]. Similar to other lipids, cholesterol is also affected by oxidative stress, which can change the aggregation properties of the proteins residing in lipid rafts. Bosco and his co-workers detected the presence of oxidized cholesterol metabolites in Lewy bodies, and proposed that oxidative stress produces cholesterol aldehydes that may cause α-synuclein aggregation [84]. In the same direction, Giasson and colleagues identified a selective and specific nitration of α-synuclein in both PD and dementia [85], and concluded that the oxidative and nitrative damage caused by it are directly linked to neurodegeneration. As the nitration of tyrosines in the C-terminus of the protein may restrict its binding to the membrane [86], it is possible that the oxidation and nitration processes could promote synapse damage and, at the same time, release α-synuclein into the cytoplasm, where it may aggregate due to the exposure of previously hidden hydrophobic patches (Fig. 1c).

### Membrane perturbation in amyotrophic lateral sclerosis

Superoxide dismutase 1 is the best characterized gene of the proteins that lead to ALS, which is a paralytic disorder caused by the degeneration of motor neurons [87].

As described for APP, tau and α-synuclein, SOD1 also has the ability to bind to membranes [61], with a significant fraction associated with lipid rafts [88]. In fact, lipid binding and oxidation processes can enhance SOD1 oligomerization and the formation of cytotoxic aggregates [89, 90]. Accordingly, alterations in membrane dynamics may redistribute SOD1, modulating its aggregation propensity. For example, lipid peroxidation can generate radicals in the membrane that promote the oligomerization of the protein (Fig. 1d).

Other proteins have also been related to ALS, including TDP-43, alsin and dynactin. To the best of our knowledge, TDP-43 membrane-binding activities have not yet been characterized. However, alsin and dynactin play relevant roles in membrane traffic: alsin plays a part in cell membrane organization and endocytosis, while dynactin is essential for vesicle movement [91, 92].

### Membrane perturbation in Huntington’s disease

Huntingtin is a large protein that is related to development and apoptosis, and is neuropathologically linked to HD. Its aggregation is due to the presence of a poly-Q repeat, which is formed either by the configuration of hydrogen bonds between amide residues or through the action of transglutaminase [93]. It is believed that huntingtin aggregates may cause neurodegeneration by sequestering other essential proteins and disrupting the protein quality control machinery [94].

Huntingtin is normally associated with membranes by palmitoylation, which is essential for its function and traffic inside the cell [95]. Bertoni and colleagues observed that an expansion of the poly-Q repeat causes the migration of the protein to lipid rafts, where it interacts with gp91, a membrane NADPH-oxidase subunit. Gp91, in turn, stimulates ROS production and DNA damage [96]. Additionally, the poly-Q aggregates are also able to induce membrane damage [97].

## Future perspectives

From the literature examined in this paper, it is reasonable to conclude that perturbations at the membrane level may have a major impact on the folding, location and function of proteins and a direct implication in disease. Furthermore, membrane perturbation can also trigger functional changes in membrane-anchored proteins [98] that may be relevant for the progression of a disorder, including G-coupled receptors that can alter Aβ production and degradation [99, 100].

**Fig. 2:**
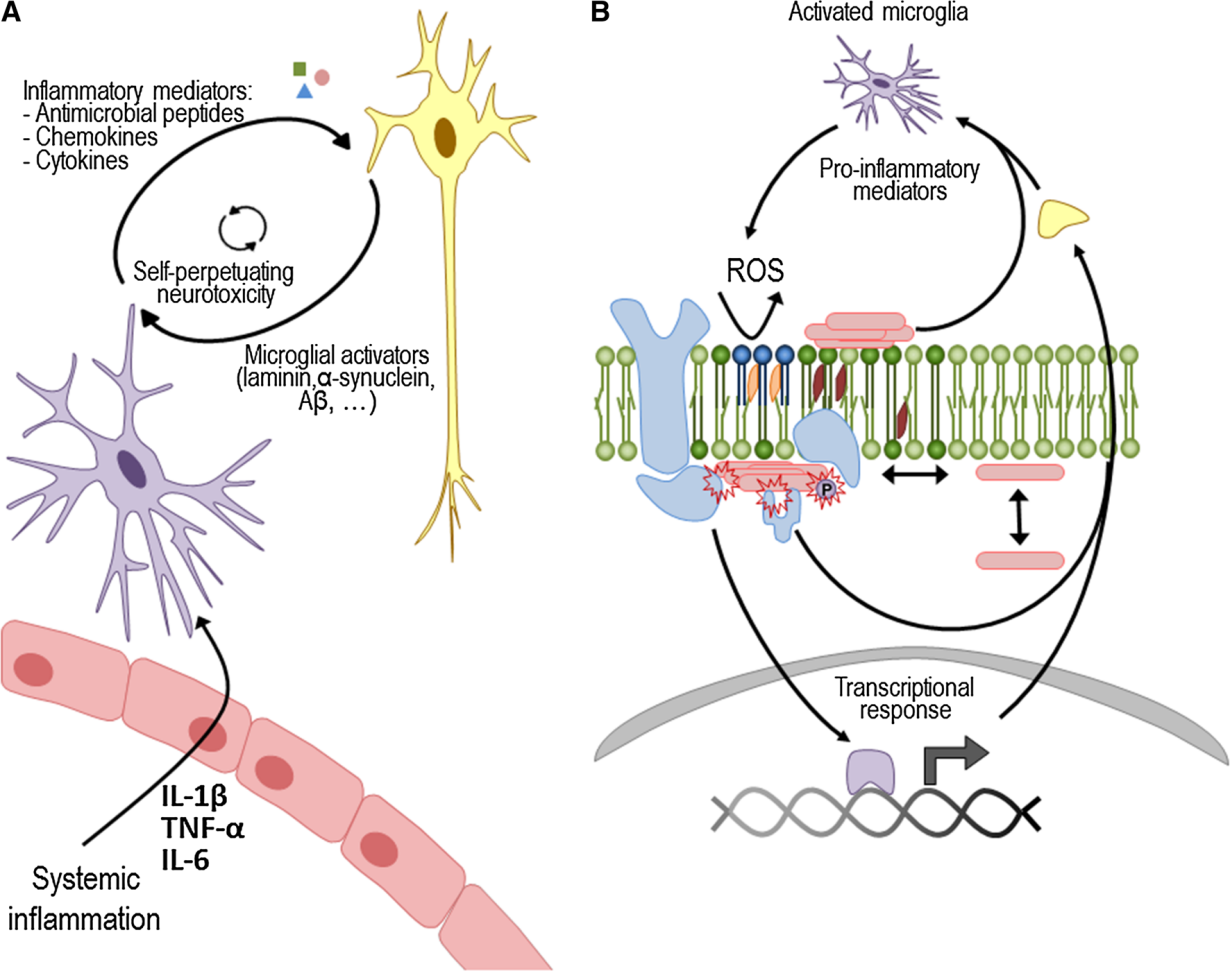
Membrane damage as an explanation on how microglia-secreted mediators may promote protein aggregation. **a** When a systemic infection is detected in the body, several inflammatory mediators are released. Some of these can cross the blood–brain barrier and stimulate microglial cells (*purple*). These cells change its morphology and become activated, releasing pro-inflammatory cytokines, ROS and other peptide mediators in the brain. When a chronic inflammatory signal is present, microglia cells become permanently primed, and the continued release of pro-inflammatory mediators can damage the surrounding neuronal cells (*yellow*). In turn, neuronal damage contributes to increasing the microglia activators that further activate microglial cells, causing a dangerous, self-sustaining activation cycle. **b** At the molecular level, ROS and the binding of pro-inflammatory mediators to specific receptors generate a transcriptional response in neurons. Moreover, ROS can cause lipid peroxidation (*dark blue*), thus perturbing neuronal membrane homeostasis, while the unspecific binding of peptide mediators can alter membrane fluidity. Globally, the integrated response to microglial secretions at the membrane level may lead to aggregation and protein mislocation. This may also trigger changes in post-translational modifications, including the phosphorylation state of several proteins such as α-synuclein, Aβ and tau. Last but not least, protein aggregates themselves or through the activation of receptors and signaling complexes (e.g. TLR2 or NLRP3 inflammasome) may generate a self-perpetuating mechanism of increased membrane damage and aggregation

Taking into consideration all the data reported above, we suggest that neuroinflammation promoted by the dysfunctional actions of microglia can cause an imbalance in membrane homeostasis, thus promoting or aggravating protein aggregation (Fig. 2). Additionally, amyloid aggregates, mainly oligomers [101], can also destabilize membranes [102] and contribute further to worsen inflammation by activating the NLRP3 inflammasome and Toll-like receptor 2 [103]. These events may, thus, generate a self-perpetuating mechanism of increased membrane damage and aggregation (Fig. 2).

If inflammation can effectively trigger or accelerate the progression of neurodegenerative diseases it would provide promising avenues for the development of new biomarkers and drugs to fight these pathologies. Several authors have already proposed that NLRP3 inflammasome inhibition represents a new therapeutic intervention for the disease [104]. In fact, small molecules have been designed to inhibit the NLRP3 inflammasome for the treatment of inflammatory diseases [105]. All these results are encouraging and bring us closer to an effective treatment for neurodegenerative pathologies.

It is our view that comprehension of the link between inflammation, membrane homeostasis and protein conformational plasticity will lead to a better understanding of diseases. Consequently, a multidisciplinary approach is required:

(1) At the biochemistry and biophysics level to understand how membrane interaction drives the conformational switch of proteins.

(2) From a cell biology perspective to comprehend how changes in membrane dynamics and composition are translated into signaling processes and protein post-translational modifications.

(3) From an immunology point of view to identify and characterize the cells and mediators responsible for this membrane homeostasis imbalance. The results provided by future studies in these areas will hopefully lead to the development of new drugs and treatments for neurodegenerative diseases.

Last but not least, a more detailed comprehension of inflammation in less prevalent diseases (e.g., HD, ALS) may help to understand common as well as particular molecular mechanisms involved in neurodegeneration.
